# Supplementary figures and images for: Preparation and characterization of gelatin-polysaccharide composite hydrogels for tissue engineering
Source: PeerJ. 2021 Mar 15;9:e11022. doi: 10.7717/peerj.11022 (PMC7971083; doi:10.7717/peerj.11022)

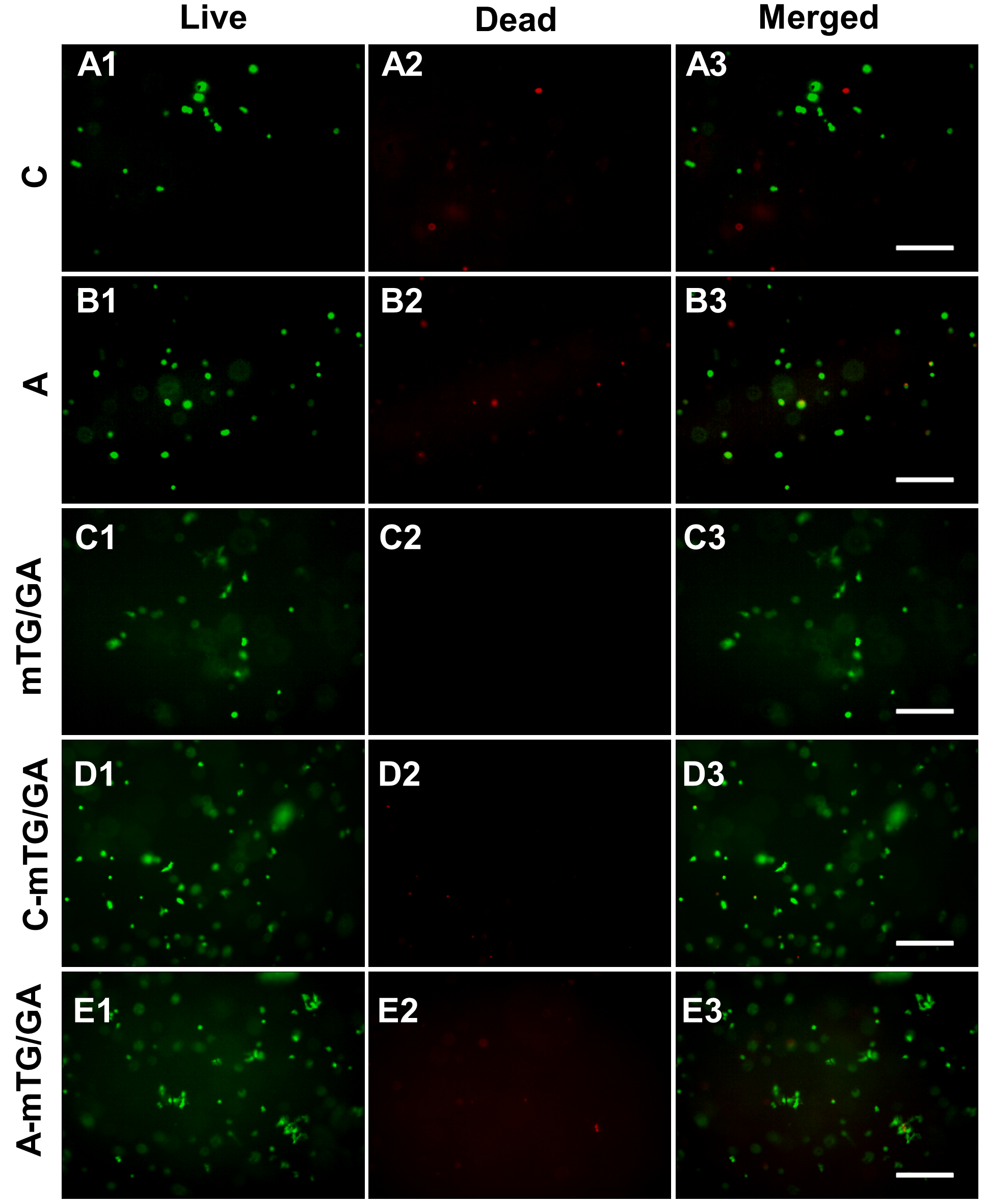

Supplement: Supplemental Information 5 — Raw data of supplementary experiment (Live/dead staining images of 3D cultures and results of degradation test). [file peerj-09-11022-s005.zip › 3Dculture_live_death_staining/Figure5.png]

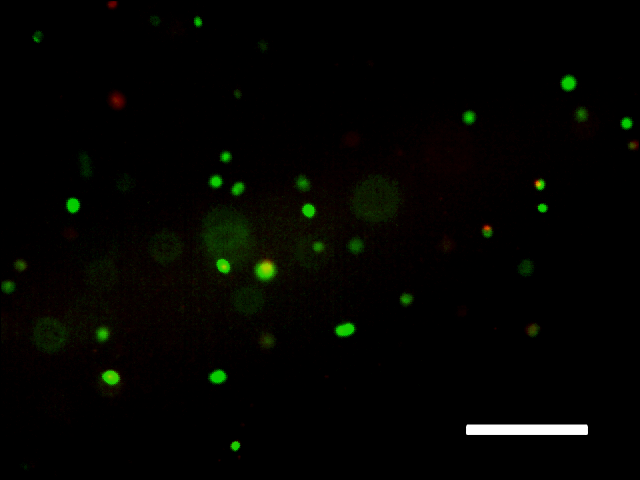

Supplement: Supplemental Information 5 — Raw data of supplementary experiment (Live/dead staining images of 3D cultures and results of degradation test). [file peerj-09-11022-s005.zip › 3Dculture_live_death_staining/Enhanced images/A.tif]

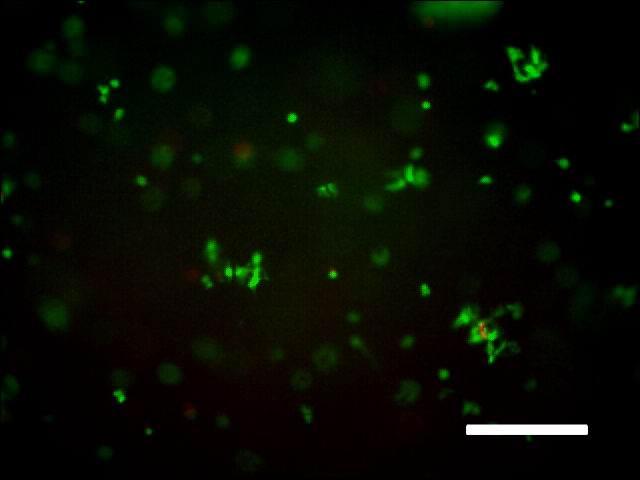

Supplement: Supplemental Information 5 — Raw data of supplementary experiment (Live/dead staining images of 3D cultures and results of degradation test). [file peerj-09-11022-s005.zip › 3Dculture_live_death_staining/Enhanced images/AGA.tif]

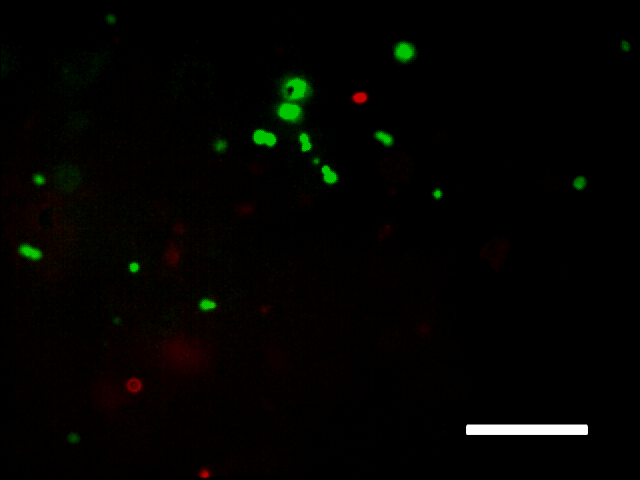

Supplement: Supplemental Information 5 — Raw data of supplementary experiment (Live/dead staining images of 3D cultures and results of degradation test). [file peerj-09-11022-s005.zip › 3Dculture_live_death_staining/Enhanced images/C.tif]

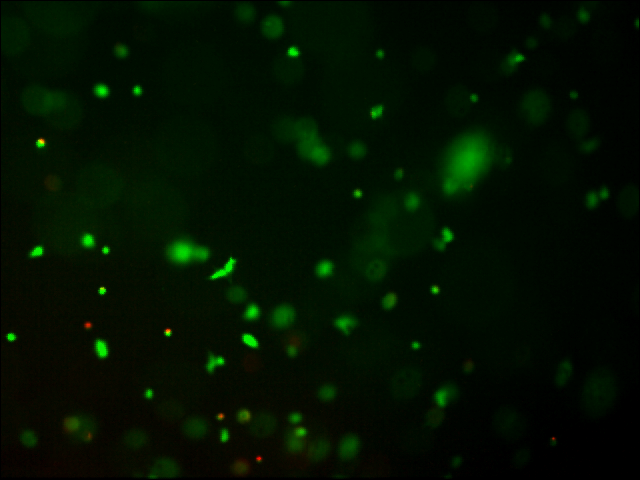

Supplement: Supplemental Information 5 — Raw data of supplementary experiment (Live/dead staining images of 3D cultures and results of degradation test). [file peerj-09-11022-s005.zip › 3Dculture_live_death_staining/Enhanced images/CAG.tif]

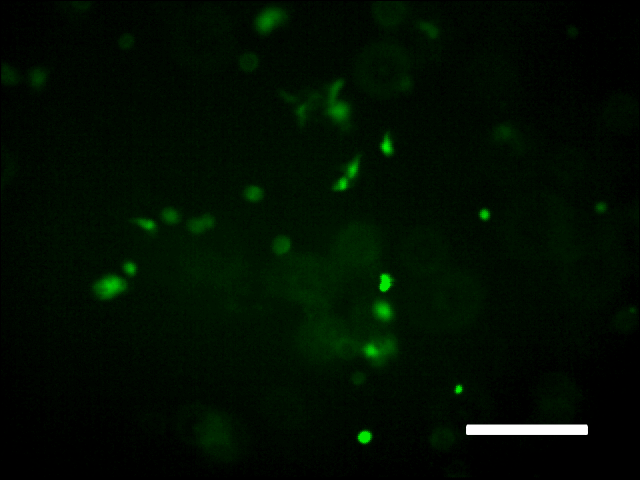

Supplement: Supplemental Information 5 — Raw data of supplementary experiment (Live/dead staining images of 3D cultures and results of degradation test). [file peerj-09-11022-s005.zip › 3Dculture_live_death_staining/Enhanced images/GA.tif]

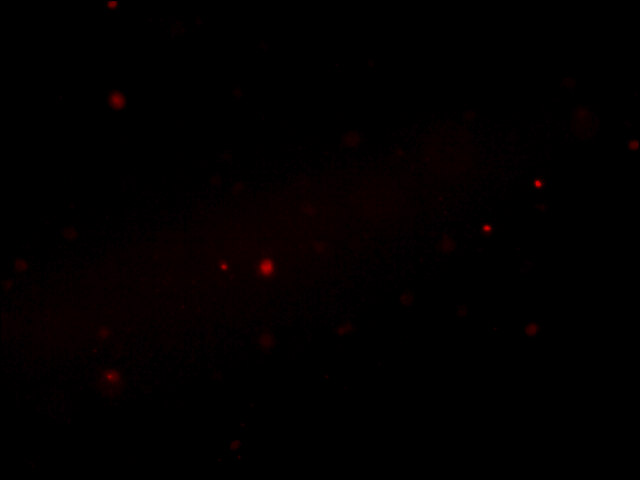

Supplement: Supplemental Information 5 — Raw data of supplementary experiment (Live/dead staining images of 3D cultures and results of degradation test). [file peerj-09-11022-s005.zip › 3Dculture_live_death_staining/Enhanced images/A_Dead.tif]

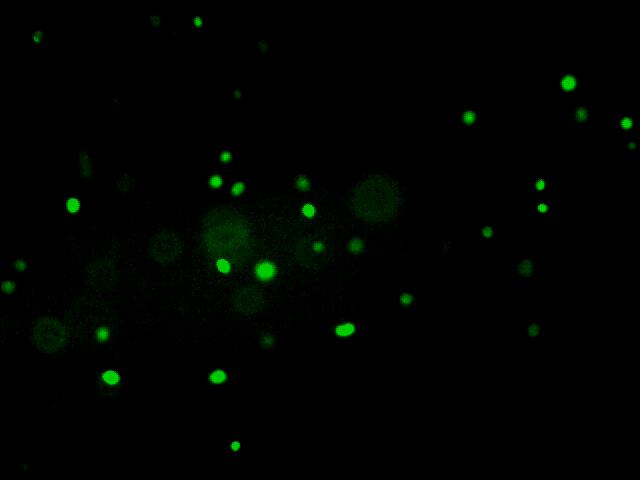

Supplement: Supplemental Information 5 — Raw data of supplementary experiment (Live/dead staining images of 3D cultures and results of degradation test). [file peerj-09-11022-s005.zip › 3Dculture_live_death_staining/Enhanced images/A_Live.tif]

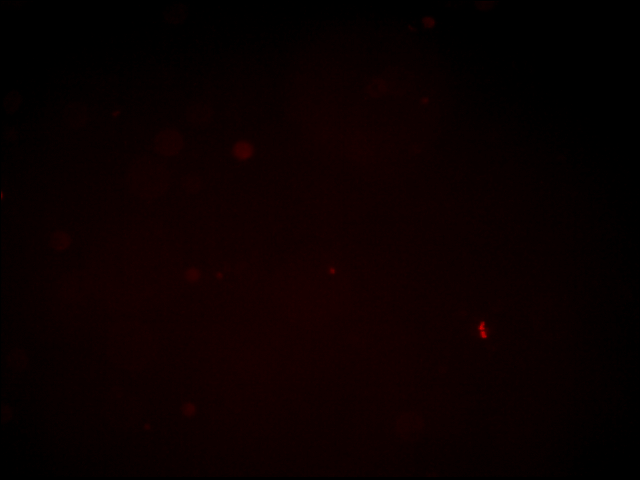

Supplement: Supplemental Information 5 — Raw data of supplementary experiment (Live/dead staining images of 3D cultures and results of degradation test). [file peerj-09-11022-s005.zip › 3Dculture_live_death_staining/Enhanced images/AGA_Dead.tif]

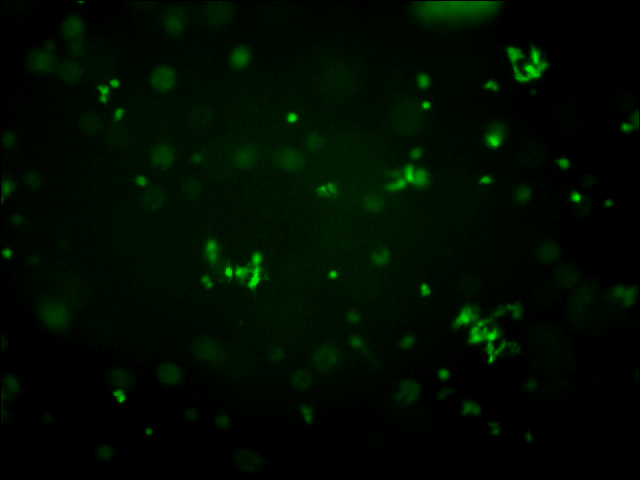

Supplement: Supplemental Information 5 — Raw data of supplementary experiment (Live/dead staining images of 3D cultures and results of degradation test). [file peerj-09-11022-s005.zip › 3Dculture_live_death_staining/Enhanced images/AGA_Live.tif]

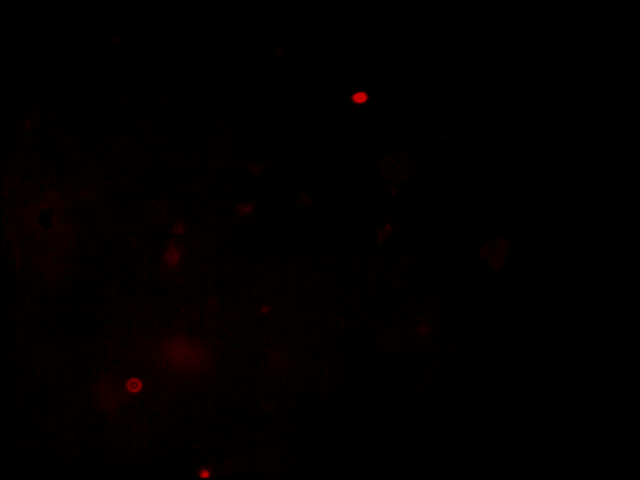

Supplement: Supplemental Information 5 — Raw data of supplementary experiment (Live/dead staining images of 3D cultures and results of degradation test). [file peerj-09-11022-s005.zip › 3Dculture_live_death_staining/Enhanced images/C_Dead.tif]

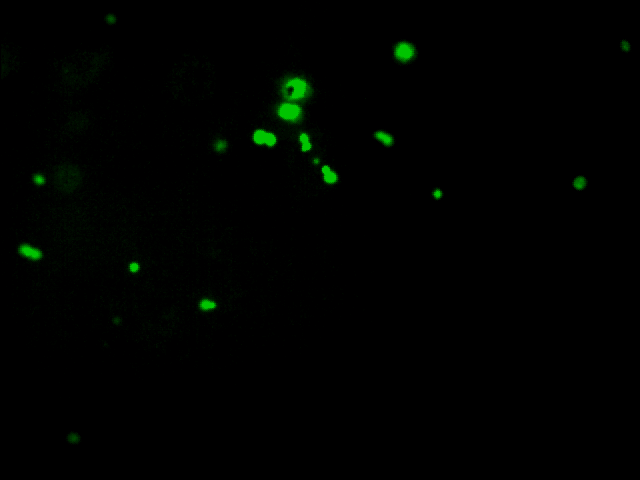

Supplement: Supplemental Information 5 — Raw data of supplementary experiment (Live/dead staining images of 3D cultures and results of degradation test). [file peerj-09-11022-s005.zip › 3Dculture_live_death_staining/Enhanced images/C_Live.tif]

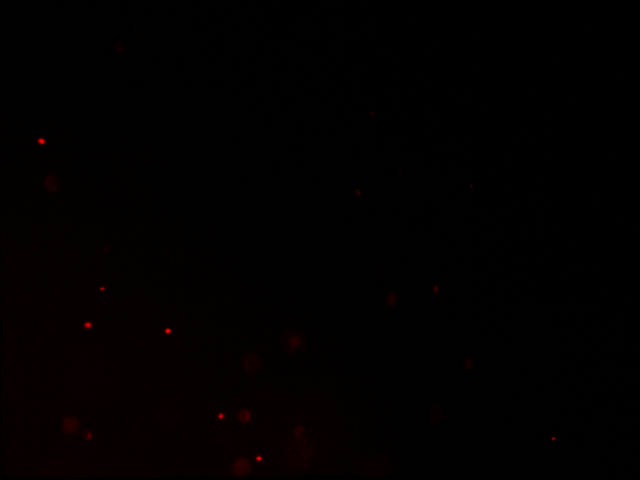

Supplement: Supplemental Information 5 — Raw data of supplementary experiment (Live/dead staining images of 3D cultures and results of degradation test). [file peerj-09-11022-s005.zip › 3Dculture_live_death_staining/Enhanced images/CAG_Dead.tif]

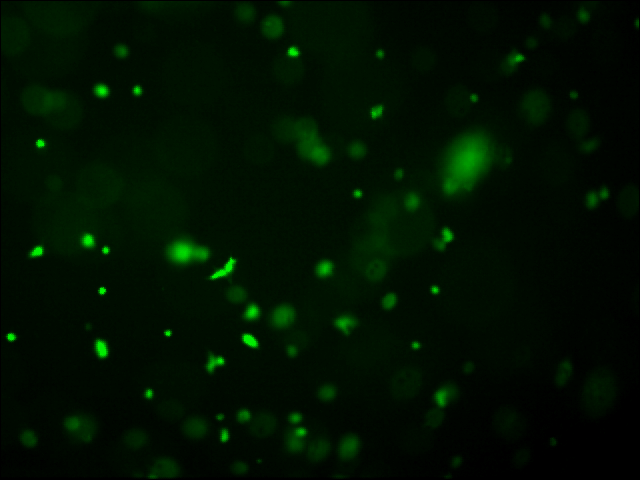

Supplement: Supplemental Information 5 — Raw data of supplementary experiment (Live/dead staining images of 3D cultures and results of degradation test). [file peerj-09-11022-s005.zip › 3Dculture_live_death_staining/Enhanced images/CAG_Live.tif]

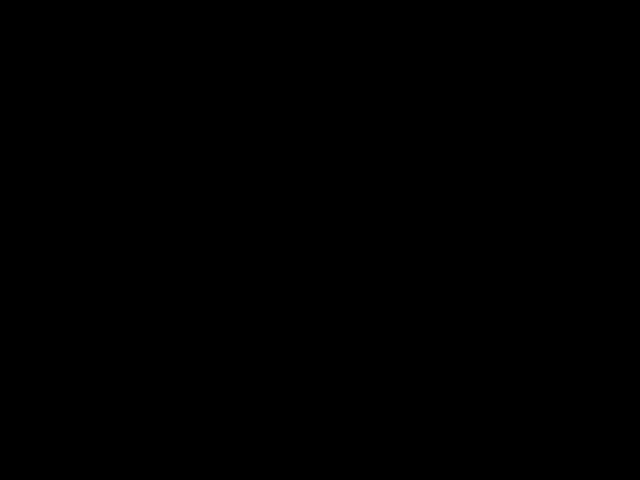

Supplement: Supplemental Information 5 — Raw data of supplementary experiment (Live/dead staining images of 3D cultures and results of degradation test). [file peerj-09-11022-s005.zip › 3Dculture_live_death_staining/Enhanced images/GA_Dead.tif]

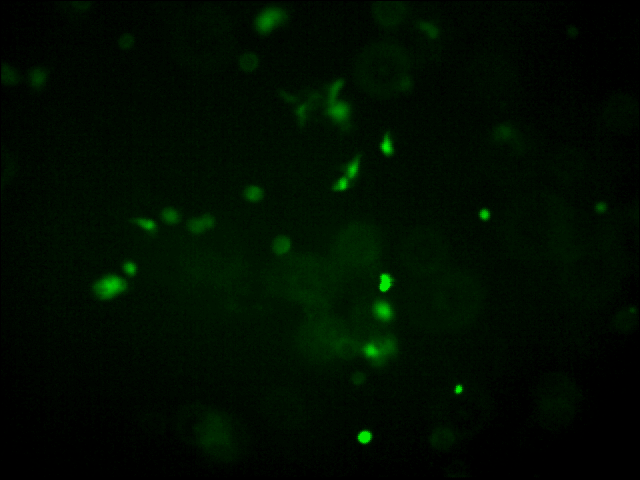

Supplement: Supplemental Information 5 — Raw data of supplementary experiment (Live/dead staining images of 3D cultures and results of degradation test). [file peerj-09-11022-s005.zip › 3Dculture_live_death_staining/Enhanced images/GA_Live.tif]

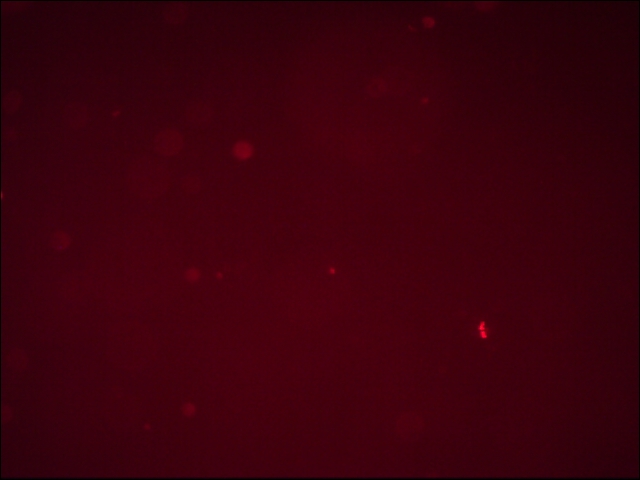

Supplement: Supplemental Information 5 — Raw data of supplementary experiment (Live/dead staining images of 3D cultures and results of degradation test). [file peerj-09-11022-s005.zip › 3Dculture_live_death_staining/Raw images/ADSCs_AmTGGA_3D_death_5d.jpg]

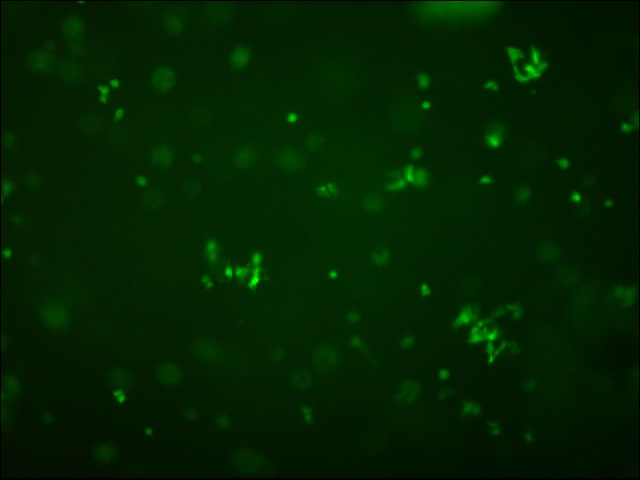

Supplement: Supplemental Information 5 — Raw data of supplementary experiment (Live/dead staining images of 3D cultures and results of degradation test). [file peerj-09-11022-s005.zip › 3Dculture_live_death_staining/Raw images/ADSCs_AmTGGA_3D_live_5d.jpg]

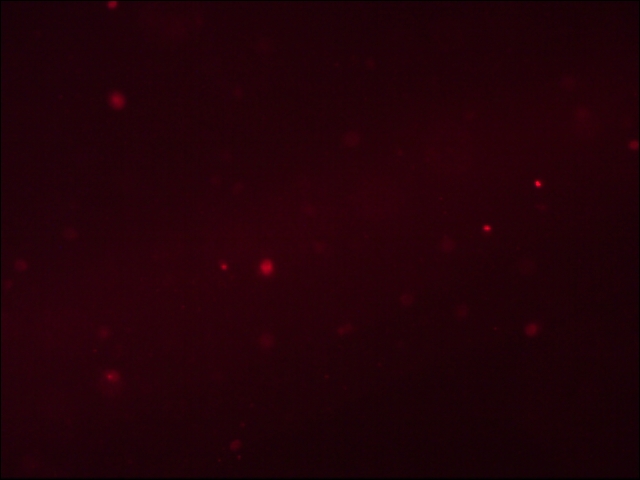

Supplement: Supplemental Information 5 — Raw data of supplementary experiment (Live/dead staining images of 3D cultures and results of degradation test). [file peerj-09-11022-s005.zip › 3Dculture_live_death_staining/Raw images/ADSCs_A_3D_death_5d.jpg]

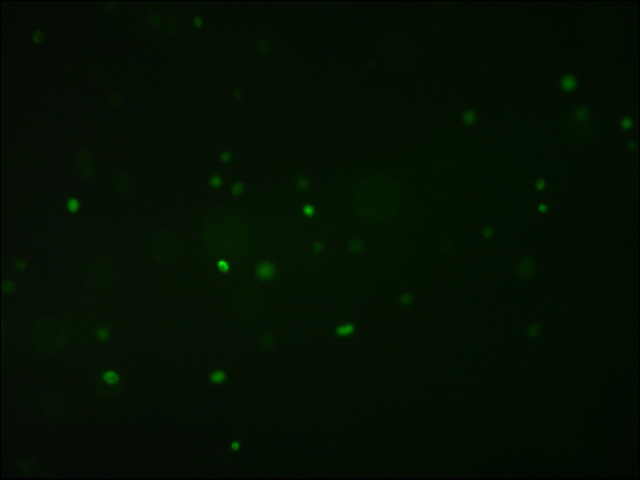

Supplement: Supplemental Information 5 — Raw data of supplementary experiment (Live/dead staining images of 3D cultures and results of degradation test). [file peerj-09-11022-s005.zip › 3Dculture_live_death_staining/Raw images/ADSCs_A_3D_live_5d.jpg]

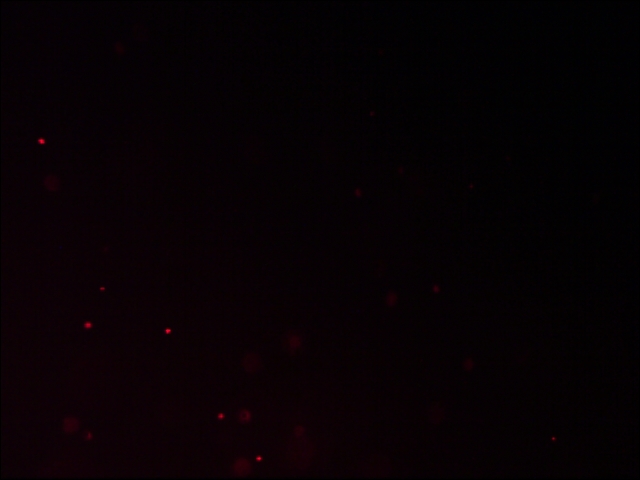

Supplement: Supplemental Information 5 — Raw data of supplementary experiment (Live/dead staining images of 3D cultures and results of degradation test). [file peerj-09-11022-s005.zip › 3Dculture_live_death_staining/Raw images/ADSCs_CmTGGA_3D_death_5d.jpg]

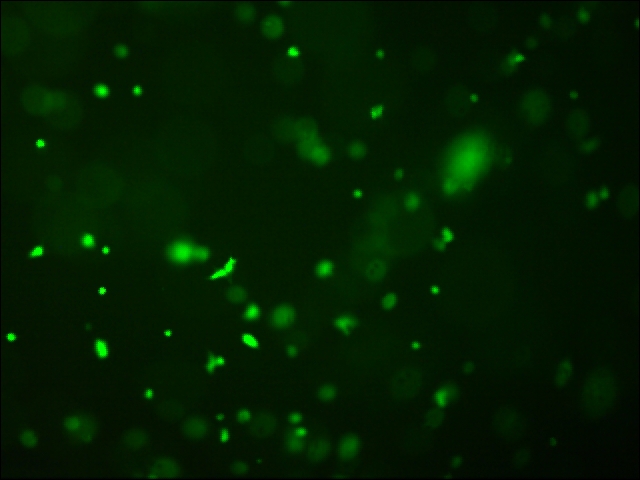

Supplement: Supplemental Information 5 — Raw data of supplementary experiment (Live/dead staining images of 3D cultures and results of degradation test). [file peerj-09-11022-s005.zip › 3Dculture_live_death_staining/Raw images/ADSCs_CmTGGA_3D_live_5d.jpg]

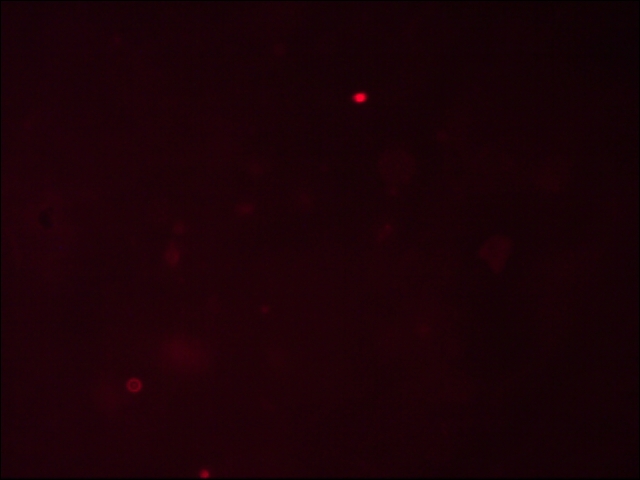

Supplement: Supplemental Information 5 — Raw data of supplementary experiment (Live/dead staining images of 3D cultures and results of degradation test). [file peerj-09-11022-s005.zip › 3Dculture_live_death_staining/Raw images/ADSCs_C_3D_death_5d.jpg]

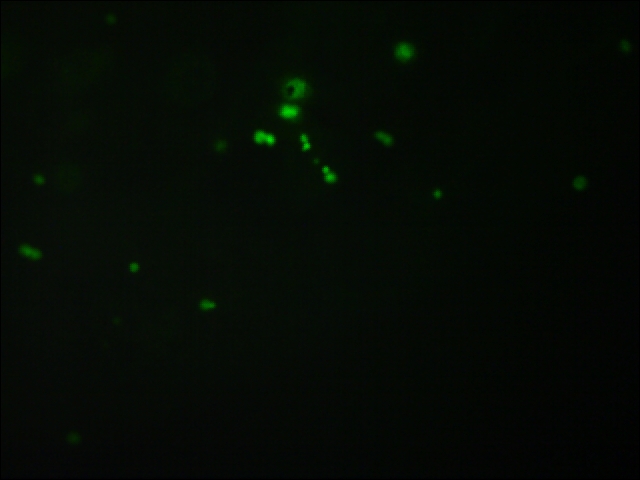

Supplement: Supplemental Information 5 — Raw data of supplementary experiment (Live/dead staining images of 3D cultures and results of degradation test). [file peerj-09-11022-s005.zip › 3Dculture_live_death_staining/Raw images/ADSCs_C_3D_live_5d.jpg]

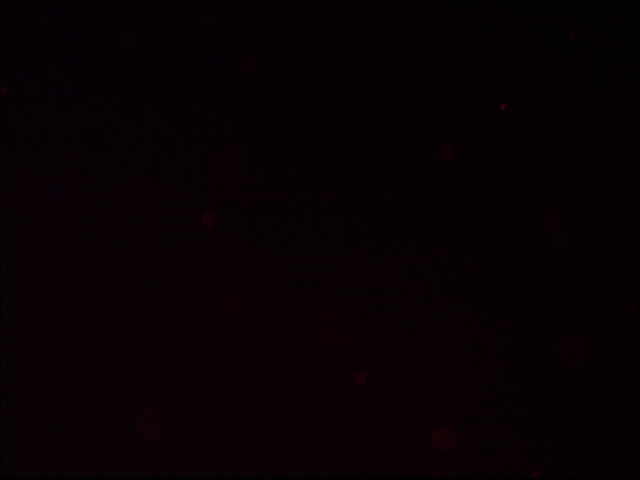

Supplement: Supplemental Information 5 — Raw data of supplementary experiment (Live/dead staining images of 3D cultures and results of degradation test). [file peerj-09-11022-s005.zip › 3Dculture_live_death_staining/Raw images/ADSCs_mTG_3D_death_5d.jpg]

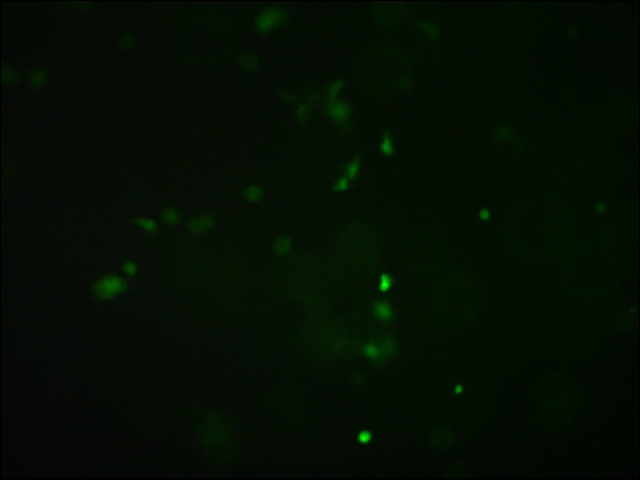

Supplement: Supplemental Information 5 — Raw data of supplementary experiment (Live/dead staining images of 3D cultures and results of degradation test). [file peerj-09-11022-s005.zip › 3Dculture_live_death_staining/Raw images/ADSCs_mTG_3D_live_5d.jpg]
